# Endovascular treatment of aorto-iliac occlusive disease with TASC II C and D lesions: 10 year’s experience of clinical technique

**DOI:** 10.1186/s12872-023-03059-4

**Published:** 2023-02-07

**Authors:** Xiangjun Dong, Ziqian Peng, Yanqiao Ren, Lei Chen, Tao Sun, Yangbo Su, Huimin Liang, Chuansheng Zheng

**Affiliations:** 1 Department of Radiology, Union Hospital, Tongji Medical College, Huazhong University of Science and Technology, Wuhan, 430022 China; 2 Hubei Key Laboratory of Molecular Imaging, Wuhan, 430022 China; 3 Department of Radiology, Yingshan People’s Hospital, Huanggang, 438700 Hubei China

**Keywords:** Aorto-iliac occlusive disease, TASC C and D lesions, Endovascular procedures, Primary patency, Intervention experience

## Abstract

**Objectives:**

The purpose of this study was to evaluate the therapeutic efficacy and safety of endovascular treatment aorto-iliac occlusive disease (AIOD) with TransAtlantic Inter-Society Consensus II (TASC II) C and D lesions. In addition, 10 years of experience with interventional procedures and treatment options in our center were also worthy of further discussion.

**Methods:**

Between January 2011 and December 2020, a total of 26 consecutive AIOD patients with TASC-II C and D lesions treated endovascular approach were enrolled in this study. Patients' demographic and clinical data were collected, and the safety and efficacy of endovascular therapy were evaluated. In addition, operation procedures were also described.

**Results:**

The mean age of patients was 62.2 ± 7 years (49–57 years), and the mean body mass index of patients was 24.2 ± 2.6 kg/m^2^. Fifteen patients (57.7%) were Rutherford 4, 5 each (19.2%) were Rutherford 3 and 5, and 1 (3.8%) was Rutherford 2. No other serious complications occurred except death in 3 patients. Most of the patients (73.1%) had a history of smoking, and hypertension and hyperlipidemia were common comorbidities. Endovascular therapy was successfully performed in 25 patients, and the technical success rate was 96.2%. The patient's ankle-brachial index improved significantly postoperatively compared with preoperatively (preoperative 0.33 ± 0.14 vs 1.0 ± 0.09, *P* < 0.001). The primary patency rates were 100%, 95.7%, and 91.3% at 1, 3, and 5 years, while the secondary patency rates were 100%. No treatment-related deaths or serious complications occurred.

**Conclusions:**

Endovascular treatment of AIOD patients with TASC-II C and D lesions might be safe and have a high rate of middle-term and long-term primary patency.

## Introduction

Aortoiliac occlusive disease (AIOD) presented an ischemic disease of the lower limbs and/or pelvic tissues and organs caused by narrowing or occlusion of the abdominal aorta beneath the renal artery and the iliac artery. Intermittent claudication is the most common clinical manifestation and can cause severe ischemia in the lower extremities if the disease continues to worsen. Therefore, appropriate treatment is urgently needed to maintain vascular patency and improve the clinical symptoms of AIOD patients.

Aortobifemoral bypass has long been the standard reconstructive procedure in vascular surgery for AIOD [1]. However, in recent years, with the development of endovascular technology and equipment, the improvement of operators' experience, as well as the pursuit of minimally invasive treatment by patients and operators, endovascular approach has gradually become the preferred method for the treatment of AIOD [2]. Meanwhile, studies have indicated that from 1996 to 2000, endovascular therapy for AIOD increased by 850% and the use of aortobifemoral bypass decreased by 15.5% [3]. In 2007, the TransAtlantic Inter-Society Consensus for the Management of Peripheral Arterial Disease (TASC II) suggested that AIOD patients classified as TASC C and D receive surgical treatment, and patients classified as A and B receive the endovascular approach [4].

Given that patients with TASC C and D often have multiple comorbidities and are not suitable for open surgery, practitioners are increasingly preferring an endovascular approach regardless of the type of lesion [5]. After all, the primary concern for high-risk patients is the ability to withstand surgical trauma, and the goal of treatment should be to effectively alleviate the severe ischemia that threatens limb survival. Based on this, many studies [6, 7] have reported endovascular treatment of TASC-II C and D lesions, in a meta-analysis [8], endovascular intervention was associated with a lower 30-day mortality rate than surgery. But to our knowledge, there is no consensus on the interventional methods and treatment options for TASC-II C and D lesions. Clinically, the treatment of TASC-II C and D lesions and the selection of stents are basically based on the experience of the interventionists, and there is no uniform standard, which is detrimental to the lack of adequate training of vascular interventional radiologists and may ultimately affect the patient's prognosis.

Meanwhile, according to the latest guidelines [9], endovascular approach may be considered for TASC-II D lesions in patients with severe comorbidities, although the guidelines suggested that this should be performed by an experienced team, which also emphasizes the importance of interventional approach and experience. Therefore, this study retrospectively analyzed the clinical data of endovascular treatment of AIOD patients with TASC-II C and D lesions. In addition to the evaluation of the safety and effectiveness of endovascular therapy, we summarized the 10 years of management experience of our center, focusing on the introduction of interventional operation and treatment options, hoping to provide some references for the management of TASC-II C and D lesions.

## Materials and methods

### Study design and patient selection

This retrospective observational study was approved by the local hospital ethics committee. Writtern informed consent was waived for this retrospective study. In this cohort study at our institution, medical records of patients who received endovascular treatment between January 2011 and December 2020 were retrospectively analyzed. Inclusion criteria [6] were AIOD patients with symptomatic chronic complete aorta, iliac or aortic iliac occlusion and chronic limb ischemia with secondary thrombosis (> 2 months). Exclusion criteria were AIOD patients undergoing open surgery, patients with aortic or iliac artery dissection resulting in occlusion, or patients with vascular stenosis/occlusion following repair of an aneurysm. According to the above inclusion and exclusion criteria, a total of 26 patients receiving endovascular treatment were included in this study. All the experiments in this study were conducted in accordance to the Declaration of Helsinki.

### Angiography and endovascular treatment

A team of vascular specialists, including interventional radiologists and vascular surgeons, determines whether an endovascular approach is appropriate for each patient. Meanwhile, the treatment procedure was determined by taking into account the morphology of the lesion, the patient's comorbidities and life expectancy, and the patient's preferences.

All operations are performed under local anesthesia and the modified Seldinger technique was adopted. The patient's bilateral femoral arteries were first punctured. When the 0.035-in. guide wire passes through the occlusion segment, the balloon is first introduced for expansion. It should be noted that in order to prevent the rupture of blood vessels, a balloon with a small diameter must be used. In our center, the balloon with a diameter of 5–6 mm is basically used. After predilation, the stent is inserted into the occlusion segment under the guidance of the guide wire. Stent selection is based on the patient's angiography. Some patients used kissing stent technique to place two stents, and some patients used the combined stent method to place one aortic stent and two aortoiliac kissing stents. Regarding the choice of bare stents and covered stents, we mainly made the decision based on the patient's angiography. Meanwhile, in patients at risk of rupture of the aortic or iliac arteries, the covered stent should be placed.

Patients were routinely given anticoagulant therapy after endovascular therapy, namely subcutaneous injection of low molecular weight heparin (5000 IU/12 h) for 3–5 days. After that, clopidogrel (75 mg/d) and aspirin (100 mg/d) were given for antiplatelet therapy, followed by aspirin alone (100 mg/d) 3 months later.

### Definition and evaluation of data

The TASC II classification [4] was applied to characterize the categories of aortoiliac lesions, and the Rutherford category was employed to grade the severities of ischemia [10]. Technical success was defined as a residual stenosis of less than 30%, a pressure gradient of less than 10 mmHg, and no distal emboli or vessel perforation. Primary patency was defined as uninterrupted patency of the treated lesion during follow-up. Primary assisted patency was defined as patency of the treated segment following endovascular approach at the lesion site in case of symptomatic restenosis, but without occlusion at any time. Secondary patency was defined as restored blood flow through the originally target lesion [2, 11]. Perioperative death was defined as death within 30 days of receiving endovascular treatment. Complications were assessed according to the Society of Interventional Radiology [12]. Major complications were defined as events leading to death and disability that increase the level of care, or result in hospital admission, or substantially lengthen the hospital stay.

### Assessment and follow-up

Demographic, clinical and perioperative data were collected. In addition, perioperative ankle-brachial index (ABI) was collected to assess the effects of endovascular approach. Significant restenosis was defined by a focal increase in peak systolic velocity (PSV) greater than 300 cm/s, a PSV ratio greater than 3.0, and uniform PSV less than 50 cm/s throughout the stent [13]. Follow-up included ultrasound and/or computer tomography angiography (CTA) examinations at 3, 6, and 12 months in the first year and annually thereafter to assess the patency of the treated vessels. The follow-up time was 3–60 months, with an average of 26.8 ± 19.3 months.

### Statistical analysis

SPSS software (version 24.0; IBM, Armonk, New York) was used for statistical analysis. Continuous data were represented by means ± standard deviations and discrete variables were represented by proportion. We compared quantitative variables using the t-test.

## Results

### Study population and patient characteristics

From January 2011 to December 2020, a total of 26 AIOD patients with TASC-II C and D lesions were included in this study, including 22 males and 4 females, with an average age of 62.2 ± 7 years (range, 49–75 years). Most of the patients (73.1%) had a history of smoking, and hypertension and hyperlipidemia were common comorbidities. The mean BMI was 24.2 ± 2.6 kg/m^2^. Fifteen patients (57.7%) were Rutherford 4, 5 each (19.2%) were Rutherford 3 and 5, and 1 (3.8%) was Rutherford 2. The detailed clinical characteristics of the 26 patients are shown in Table 1.

**Table 1:** Baseline clinical characteristics of patients

Characteristic	AIOD Patients with TASC-II C and D lesions (No, %; Mean ± SD)
Gender	
Male	22 (84.6%)
Female	4 (15.4%)
Age (y)	62.2 ± 7
Body mass index, kg/m^2^	24.2 ± 2.6
Preoperative ABI	0.33 ± 0.14
Rutherford category	
1–3	6 (23.1%)
4–6	20 (76.9%)
TASC lesion type	18.9 ± 17.2
C	6 (23.1%)
D	20 (76.9%)
Comorbidities	
Hypertension	9 (34.6%)
Diabetes mellitus	6 (23.1%)
Dyslipidemia	5 (19.2%)
Coronary artery disease	3 (11.5%)
Smoking	19 (73.1%)
Cerebrovascular disease	3 (11.5%)
Hemoglobin (g/dL)	133.8 ± 11.7
Albumin (g/dL)	38.3 ± 3.1
Creatinine (mg/dL)	83.1 ± 12.4
BUN (mmol/L)	4.4 ± 0.7
GFR (mL/min/1.73m^2^)	88.2 ± 12.2
Puncture approach	
Bilateral femoral artery	5 (19.2%)
Brachial and femoral arteries	21 (80.8%)
Operation time, min	90.5 ± 33.7

SD standard deviation

### Technical and clinical outcomes

Bilateral femoral artery percutaneous puncture was performed in all patients, of whom 5 patients (19.2%) received bilateral femoral artery approach only, and 21 patients (80.8%) also had left brachial artery puncture. Endovascular therapy was successfully performed in 25 patients, and the technical success rate was 96.2%. Among them, 12 patients were treated with bare stents alone, 6 patients were treated with covered stents alone, and 7 patients were treated with bare stents combined with covered stents. A total of 89 stents were received in 25 patients who were successfully treated, with an average of 3.4 ± 1.2 stents per patient (2–5). The average operation time was 90.5 ± 33.7 min.

Of the 25 patients who received successful endovascular therapy, no reintervention, thrombosis, or death occurred within 30 days after operation. The mean ABI was significantly improved postoperatively compared with preoperatively (preoperative 0.33 ± 0.14 vs 1.0 ± 0.09, *P* < 0.001). All patients underwent routine follow-up visits after endovascular treatment, with a mean follow-up time of 26.8 ± 19.3 months (3–60 months). A total of 5 patients were lost to follow-up, including 1 patient lost to follow-up after 1 year, 2 patients lost to follow-up after 2 years, and 2 patients lost to follow-up after 3 years. During follow-up, 2 patients died and 2 patients developed restenosis, with a restenosis rate of 8%. The primary patency rates were 100%, 95.7%, and 91.3% at 1, 3, and 5 years, while the secondary patency rates were 100%.

### Complications

No serious complications such as death occurred during the perioperative period. Common minor complications included hematoma at the puncture site in 3 patients, hip pain in 13 patients, and fever in 5 patients. All hematomas at the puncture site were resolved conservatively, and symptoms of pain and fever were significantly improved after symptomatic management.

## Discussion

To our knowledge, this is the first contemporary study to focus on endovascular operation modalities for the treatment of TASC II C and D lesions. In addition, all operations were performed by the same team of experienced interventional radiologists in a single academic institution.

According to the recommendations of the TASC II [4], which is internationally recognized, surgery is the preferred treatment for types C and D lesions, while endovascular methods are appropriate for types A and B lesions. However, there are also reports of endovascular treatment of type C and D lesions [6, 7]. However, there has been no report on the technical difficulties of endovascular treatment, which is crucial for the successful treatment of type C and D lesions. After all, many patients cannot tolerate surgical operations. In this meta-analysis[8], which compared covered endovascular reconstruction of aortic bifurcation (CERAB), open surgery (OS), and standard endovascular therapy standard endovascular treatments (SEV), it was found that while OS had higher primary patency rates at 1 and 3 years, secondary patency rates were similar, and 30-day mortality was lower after endovascular intervention. Therefore, how to successfully treat type C and D lesions is a necessary skill for interventional radiologists and vascular surgeons.

Currently, there is no uniform standard for the choice of puncture approach. Proper puncture approach can reduce the operation time and facilitate true lumen re-entry. Although Shen et al. [6] and Nanto et al. [14] both mentioned that brachial artery puncture can be performed when necessary, it was only mentioned in passing and did not specify the circumstances in which brachial artery puncture was necessary. In our experience, in patients with TASC II D lesions, left brachial artery puncture is necessary to largely avoid serious complications such as aortic dissection or further renal artery involvement by dissection. In this study, 1 AIOD patient with TASC II D lesions received endovascular treatment in a local hospital. Due to the inexperience of doctors in the local hospital, only the patient's bilateral femoral arteries were punctured, and then the guide wire was attempted to enter the true lumen of the aorta, but it failed to cause aortic and renal artery dissection at the same time. In addition, the ultimate goal of endovascular therapy is to send the guide wire into the true vascular lumen and open the occluded blood vessels. For the situation that neither retrograde nor anterograde approaches can make the guide wire enter the true lumen, “docking” technology may be one of the solutions.

**Fig. 1:**
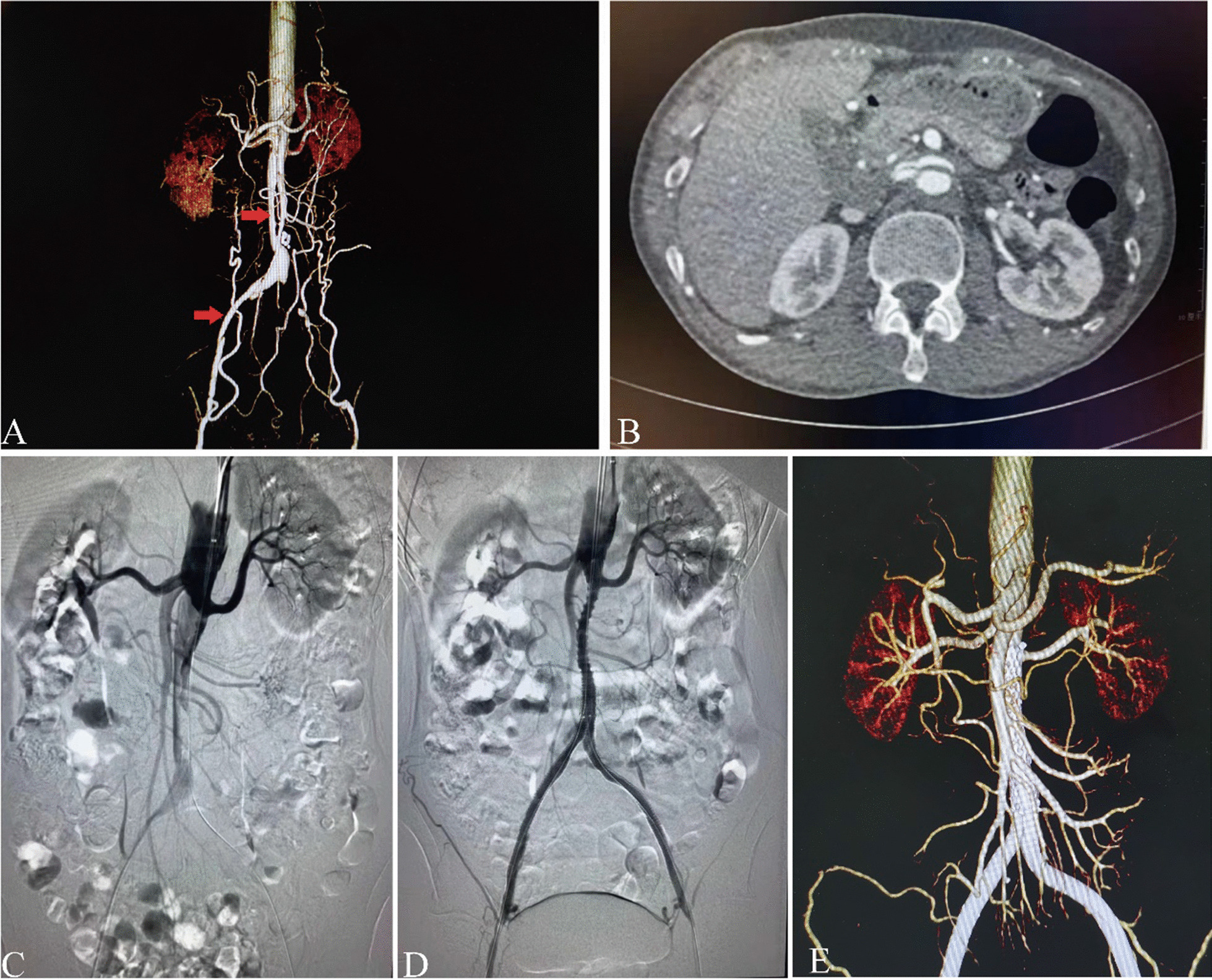
A 57-year-old male patient presented to a local hospital with intermittent claudication. After examination at the local hospital, the patient was diagnosed with AIOD with TASC II D lesion and received endovascular treatment at the local hospital. However, due to the limited technical level of the local hospital, the operation was not successful, so the patient was urgently transferred to our center. Preoperative CTA indicated the dissection of the patient's right external iliac artery abdominal aorta (**A**, shown by the red arrow), and right renal artery (**B**). The patient was transferred to our department for emergency angiography and stent implantation, and the operation was very successful (**C**, **D**). The stent position and patency were well demonstrated by reexamination 3 months after the operation (**E**)

Also, in our experience, in patients with TASC-II D, left brachial artery access is necessary because it significantly saves operative time and facilitates true lumen re-entry, and may even prevent serious complications such as aortic dissection or further renal artery involvement by dissection (Fig. 1). For patients with TASC II C, bilateral femoral artery puncture may be sufficient. In addition, for a small number of patients, no matter the anterograde approach or the retrograde approach, the guide wire cannot successfully pass the occlusion segment. Therefore, according to our experience, the "docking" technique may be able to provide some help. Specifically, the anterograde and retrograde guidewires always have a place in the iliac artery where they can meet and then introduce a catheter through the anterograde guidewire, and then manipulate the retrograde guidewire into the catheter, so that the guide wire successfully passes through the occlusion segment. Even, we can introduce a small diameter balloon (4 mm) through the guide wire of the anterograde and retrograde approach, and then expand the balloon to expand the blood vessel where the two guide wires meet, so that the guide wire can pass through the occlusion section smoothly. It is important to note, however, that in this case the covered stent must be placed in the occlusion segment where the balloon dilates.

Careful and meticulous angiography and reasonable selection of stents based on the results of angiography may explain the high technical and clinical success rates in the present study. In this study, 25 patients were successfully treated with endovascular therapy, and the technical success rate was 96.2%, which was similar to that reported in other studies [14, 15]. Nevertheless, considering that all the patients in this study are AIOD patients with TASC II C and D lesions, it is not easy to achieve such a high technical success rate due to high requirements for endovascular operation and great technical difficulty. Similar to other studies [2, 16], significant improvement can be seen in the patients of this study in terms of the postoperative ABI, much better than the preoperative ABI.

Compared with open surgery, endovascular treatment is a less invasive and less risky approach, with lower perioperative mortality (1%-4.8%) and morbidity (1%-14%) reported [17–19]. In this study, no patients died perioperatively, and no reintervention or thrombosis occurred within 30 days postoperatively. The long-term patency of stent is one of the most important indicators to measure the therapeutic effect. A single-center retrospective analysis of clinical data in TASC C and D AIOD patients treated with endovascular and hybrid techniques demonstrated a 3-year primary patency rate of 86.6% and a secondary patency rate of 97.7% [20]. Bjorses et al. conducted a clinical analysis of 173 patients with TASC C and D lesions treated with kissing stents, and the results indicated that the 1-year primary and secondary patency rates were 97% and 100%, respectively, and the 3-year primary and secondary patency rates were 83% and 95%, respectively [21]. Compared with these studies, both primary and secondary patency rates at 1 and 3 years were higher in TASC II C and D AIOD patients in this study.

There are some limitations to the study. First of all, due to the small number of cases, regression analysis cannot be performed in data analysis, which cannot well represent the morbidity of most patients and the changes after treatment. Like other studies in Table 2, the sample size was small but the findings were similar. The second reason for the analysis is that most of the patients are older and no longer willing to receive treatment even if complications occur. Besides, the change of contact information and other reasons have caused such a large loss of follow up rate. In the future, large sample and multi-center data will be needed to further verify our conclusion.

**Table 2:** Other studies with small sample sizes

References	Number of patients	Mean follow-up	Study type
Ali et al. [22]	21	12.4	Single centre retrospective
De Roeck et al. [23]	26	26	Single centre retrospective
Greinera et al. [24]	25	15.9 ± 9.4	Single centre retrospective
Inui et al. [25]	21		Single centre retrospective
Lagana et al. [26]	19	19.6	Single centre retrospective
van Haren et al. [27]	10	40 ± 24	Single centre retrospective

In conclusion, endovascular treatment of AIOD patients with TASC II C and D lesions is safe and effective, with high middle-term and long-term primary patency rate. In addition, this study is the first to discuss interventional therapy methods, and it is hoped that the experience of this center can provide some help for the treatment of AIOD patients.

## Data Availability

The data analysed during this study are avaliable from the electrical medical database of Union Hospital, Tongji Medical college, Huazhong University of Science and Technology. Please contact the author Chuansheng Zheng upon reasonable requests.

## Abbreviations

AIOD: Aortoiliac occlusive disease
TASC II: TransAtlantic Inter-Society Consensus II
ABI: Ankle-brachial index
PSV: Peak systolic velocity
CTA: Computer tomography angiography
